# Preprandial ghrelin is not affected by macronutrient intake, energy intake or energy expenditure

**DOI:** 10.1186/1477-5751-4-2

**Published:** 2005-03-03

**Authors:** David R Paul, Matthew Kramer, Donna G Rhodes, William V Rumpler

**Affiliations:** 1 U.S. Department of Agriculture, Agricultural Research Service, Diet and Human Performance Laboratory, Beltsville Human Nutrition Research Center, Beltsville, MD 20705, USA

## Abstract

**Background:**

Ghrelin, a peptide secreted by endocrine cells in the gastrointestinal tract, is a hormone purported to have a significant effect on food intake and energy balance in humans. The influence of factors related to energy balance on ghrelin, such as daily energy expenditure, energy intake, and macronutrient intake, have not been reported. Secondly, the effect of ghrelin on food intake has not been quantified under free-living conditions over a prolonged period of time. To investigate these effects, 12 men were provided with an *ad libitum *cafeteria-style diet for 16 weeks. The macronutrient composition of the diets were covertly modified with drinks containing 2.1 MJ of predominantly carbohydrate (Hi-CHO), protein (Hi-PRO), or fat (Hi-FAT). Total energy expenditure was measured for seven days on two separate occasions (doubly labeled water and physical activity logs).

**Results:**

Preprandial ghrelin concentrations were not affected by macronutrient intake, energy expenditure or energy intake (all P > 0.05). In turn, daily energy intake was significantly influenced by energy expenditure, but not ghrelin.

**Conclusion:**

Preprandial ghrelin does not appear to be influenced by macronutrient composition, energy intake, or energy expenditure. Similarly, ghrelin does not appear to affect acute or chronic energy intake under free-living conditions.

## Background

Ghrelin, a peptide secreted by endocrine cells in the gastrointestinal tract, is thought to play a significant role in the regulation of energy balance due to its effects on the stimulation of food intake [1,2] and weight gain [1-3] in rodents. It has been suggested that ghrelin may also play a role in meal initiation in humans, since the concentration of ghrelin increases immediately prior to a meal [4] and decreases after eating [4-6]. Furthermore, ghrelin infusions are associated with feelings of hunger and increased energy intake during a buffet-style lunch [7].

Despite the evidence indicating a role in acute food intake, little is known about the factors regulating ghrelin and its effects on long-term energy balance in humans. One hypothesis is that ghrelin secretion is up-regulated in periods of negative energy balance and down-regulated in periods of positive energy balance [8]. Since energy balance is a function of both energy intake and expenditure, ghrelin concentrations should increase or decrease with fluctuations in food intake (macronutrient composition and/or energy intake) and/or energy expenditure. In turn, increased ghrelin concentrations should be associated with higher food intake. However, the effects of daily fluctuations in food intake and energy expenditure on ghrelin have not been investigated in humans.

The purpose of the present study was to determine how changes in macronutrient composition, energy intake, and energy expenditure affect preprandial ghrelin concentrations, and ghrelin's subsequent effects on food intake.

## Results

### Body weight and composition

Ghrelin was negatively related to body fat percentage (r = -0.46, P < 0.05) and BMI (r = -0.18, P < 0.02), but not body weight (r = -0.16, P > 0.45). There were no significant body weight changes during the seven day observation periods (2)(data not shown, P > 0.40).

### Effect of treatment on macronutrient and energy intake

The composition of the treatment beverages and their contribution to daily food intake is listed in Table 1. Overall, macronutrient intake during the seven day observation periods was primarily determined by the composition of the treatment beverages (Table 2).

**Table 1:** Macronutrient composition of treatment beverages for one day, and their proportion of total daily macronutrient and energy intake during the seven day treatment periods.

	Hi-CHO	Hi-PRO	Hi-FAT
*Composition of Treatment*			
Energy (MJ/d)	2.13	2.11	2.11
Carbohydrate	113	83	8
Protein (g/d)	6	34	7
Fat (g/d)	4	4	50
*Percentage of Total Daily Intake*			
Energy (%)	17.5 ± 4.0	17.1 ± 3.2	17.8 ± 3.4
Carbohydrate (%)	25.8 ± 4.4	20.2 ± 4.3	2.5 ± 0.7
Protein (%)	4.5 ± 12.9	28.3 ± 4.8	7.7 ± 2.3
Fat (%)	5.1 ± 2.4	5.0 ± 1.8	41.5 ± 9.9

presented as means ± SD

Hi-CHO = carbohydrate treatment beverage

Hi-PRO = protein/carbohydrate treatment beverage

Hi-FAT = fat treatment beverage

**Table 2:** Effect of the treatment beverages on macronutrient and energy intake

	Hi-CHO	Hi-PRO	Hi-FAT
*Macronutrient Intake (% of daily total)*			
Carbohydrate (%)	60.4 ± 6.1^a^	56.4 ± 6.9^b^	47.0 ± 8.3^c^
Protein (%)	13.5 ± 2.3^a,c^	16.6 ± 3.2^b^	13.5 ± 3.3^c^
Fat (%)	26.3 ± 5.7^a^	26.0 ± 5.6^a^	39.1 ± 6.3^b^
*Macronutrient (g/day) and Energy (MJ/d) Intake*			
Carbohydrate (g)	450.9 ± 80.4^a^	427.3 ± 83.3^b^	347.3 ± 102.3^c^
Protein (g)	100.6 ± 22.0^a,c^	123.4 ± 19.4^b^	97.5 ± 22.3^c^
Fat (g)	91.0 ± 34.2^a^	88.8 ± 27.6^a^	126.9 ± 29.8^b^
Energy (MJ/d)	12.6 ± 2.7	12.7 ± 2.3	12.2 ± 2.5

presented as means ± SD

different letters in the row denote statistical significance (mixed model ANOVA)

Hi-CHO = carbohydrate treatment beverage

Hi-PRO = protein/carbohydrate treatment beverage

Hi-FAT = fat treatment beverage

### Energy expenditure and macronutrient intake effects on preprandial ghrelin

Average 24 hour energy expenditure (24EE; uncorrected activity log alone) was 13.9 ± 1.9 MJ/d compared to 12.6 ± 1.6 MJ/d for total energy expenditure (TEE; doubly labeled water), which is an average over-reporting of energy expenditure of 11%. Thus, our assumption that subjects would likely misreport energy expenditure and the values would require adjustment was valid.

**Figure 1:**
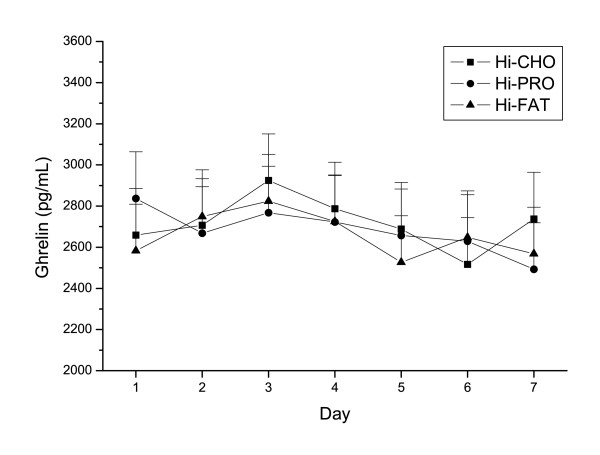
Effect of covert manipulation of macronutrient intake on preprandial ghrelin over the course of one week. Hi-CHO = carbohydrate treatment beverage Hi-PRO = protein/carbohydrate treatment beverage Hi-FAT = fat treatment beverage 1 = Monday 2 = Tuesday 3 = Wednesday 4 = Thursday 5 = Friday 6 = Saturday 7 = Sunday There were no significant treatment effects (mixed model ANOVA). Data are shown on the original scale (see text for details)

The mean preprandial ghrelin concentrations during the last week of each treatment period were 2501.4 ± 438.0 pg·mL^-1 ^for Hi-CHO, 2869.5 ± 817.3 pg·mL^-1 ^for Hi-PRO, and 2688.2 ± 755.5 pg·mL^-1 ^for Hi-FAT (Figure 1). These values are higher than reported in similar investigations. This discrepancy is explained by the use of the Linco Research Total Ghrelin RIA kit, which produces values that are approximately 10-fold higher than the most commonly used kit (Phoenix Pharmaceuticals)[9]. In a side-by-side comparison, both kits have been found to be analytically acceptable despite the differences in values obtained [9]. Furthermore, the ghrelin concentrations of at least two studies using the same kit were very similar to those we measured [10,11]. The within- and between-subject coefficients of variation for the two observation periods (seven days per period) were 12.9% and 23.0%, respectively.

Preprandial ghrelin was not influenced by treatment, 24EE, macronutrient composition, and selected (without treatment beverages) and total (including treatment beverages) energy intake (breakfast or entire day), or the interactions between these variables (previous or same day)(all P between 0.40 to 0.80). As a further test, we included energy intake for seven days prior to- and two days after each ghrelin value. None of these days were significant (all P between 0.40 to 0.90). Individual day and mean 24EE up to the prior 4 four days before each ghrelin measurement was also not significant (all P between 0.10 to 0.90).

### Effect of ghrelin and energy expenditure on macronutrient and energy intake

Selected and total energy intake for the entire day were significantly influenced by treatment period (P < 0.02), Monday/Friday effect (P < 0.003), Sunday effect (P < 0.03), and 24EE (P < 0.008) (Table 3a). Classifying energy intake into the three macronutrients, the only macronutrient influenced by 24EE was total and selected carbohydrate intake (P < 0.03, and P < 0.02, respectively) (Table 3b). There was no significant effect of ghrelin on total or selected energy intake for breakfast or entire day (all P between 0.80 to 0.90).

**Table 3:** Determinants of total energy intake (log_10_) (A) and carbohydrate intake (log_10_) (B)

A	Independant Variable	Slope	SE	P
	Intercept	-0.04	0.23	0.85
	Treatment Period	0.13	0.05	<0.02
	Sunday effect	0.18	0.08	<0.02
	Monday/ Friday effect	-0.18	0.06	<0.003
	24EE (log_10_)	1.53	0.57	<0.001

B	Independant Variable	Slope	SE	P

	Intercept	3.94	0.09	<0.0001
	Treatment Period	0.10	0.03	<0.003
	24EE (log_10_)	0.53	0.23	<0.03

Treatment Period = first 8 wk treatment vs. second 8 wk treatment period

Sunday effect = Sunday vs. other days of the week

Monday/ Friday effect = Monday and Friday vs. the other days of the week

24EE = daily energy expenditure

### Power analyses

The partial correlation between breakfast energy intake and ghrelin was 0.07. At 80% power, we could have detected a ghrelin effect if the true partial correlation was a small as 0.36. For powers of 90% and 95%, the true partial correlations would have had to be 0.40 and 0.43, respectively. The partial correlation between total energy intake and ghrelin was even lower than that with breakfast energy intake (r = 0.003). Note that, for a partial correlation of 0.40, ghrelin would have only been explaining about 16% (0.40^2^) of the variation in energy intake, still a relatively small percentage of explained variation for a hormone purported to exert a large influence on intake.

## Discussion

Of the variables related to energy balance measured in this study (daily macronutrient and energy intake, energy expenditure, and body weight and composition), none appear to play a role in preprandial ghrelin regulation. Similarly, ghrelin did not significantly predict macronutrient or energy intake, despite a power analysis indicating that we would have detected even a moderate effect of ghrelin on intake.

Most of the evidence linking food intake and ghrelin comes from single meal, short-term studies. The ingestion of amino acids or a protein meal results in a post-prandial increase in ghrelin [12-14], whereas high- [14,15] or moderate carbohydrate [4,5,16], and fat [14] meals decrease ghrelin. Carbohydrate meals may result in a greater post-prandial suppression of ghrelin than fat [16,17]. However, it has been reported that preprandial ghrelin is unrelated to macronutrient intake in a large (118 subjects) cross-sectional study [18] and a 12 week longitudinal study [19]. Similarly, three weeks of a high fat diet has been shown to have no effect on fasting ghrelin [20]. Based on the results of the current study and others [18-20], it appears that macronutrient intake does not affect preprandial ghrelin, and any macronutrient-specific effects are limited to the post-prandial period.

Wren et al.[7] were the first investigators to demonstrate that the infusion of ghrelin acutely results in an increase energy intake in humans. The lack of an energy-intake stimulating effect of ghrelin on food intake in the present study when compared to Wren et al.[7] may be related to the amount of ghrelin that was infused (resulting in concentrations twice that under fasted conditions), and the non-free living nature of the subjects. However, other studies have also failed to detect an increase in hunger after ghrelin infusion [21,22]. Ghrelin concentrations do not predict the timing of a meal request or meal size [23], and are unaffected by energy-restricted diets [10,18,24] and when appetite is increased [10]. Interestingly, it has also been shown that fasting ghrelin is negatively associated with energy intake [25]. In this same study [25], Caucasians had ghrelin concentrations that were approximately double that of Pima Indians, yet there was no difference in food intake between the groups.

Although body weight typically increases by ≈ 4.5 kg in men and ≈ 7.3 kg in women over the course of 30 years [26], the human body regulates energy balance rather well (within 1% over the course of 20 years)[27]. The strength of the relationship between total energy intake and 24EE measured in this study reflects this regulation, but our data indicate that 24EE does not influence ghrelin. One other study has shown that ghrelin does not appear to be influenced by exercise, regardless of exercise intensity [28]. This longitudinal study (three months) of normal weight young women indicated that ghrelin increases in response to an exercise regimen, but only when exercise induces weight loss. Therefore, it appears that ghrelin is not influenced by changes in energy expenditure alone.

## Conclusion

In conclusion, it appears that macronutrient and energy intake, and energy expenditure have no effect on preprandial ghrelin. None of the variables measured in this study explain the high daily variability in preprandial ghrelin observed over the course of two-seven day periods. In turn, this study fails to detect the energy intake-stimulating effect of ghrelin, despite carefully measured food intake that lasted more than a week and a study powered to detect even a moderate effect of ghrelin.

## Methods

### Subjects

Twelve healthy, non-smoking men were recruited from the Beltsville, MD area to participate in this study (Table 4). All subjects were weight-stable, and not using any medications known to affect food intake, appetite or water balance. The John Hopkins Bloomberg School of Public Health Committee on Human Research approved the study protocol. Subjects provided written informed consent and received a medical evaluation by a physician that included measurement of blood pressure and analysis of fasting blood and urine samples to screen for presence of metabolic disease.

**Table 4:** Characteristics of the subjects (n = 12)

	Mean	SD
Age (yr)	39	9
Height (m)	1.81	0.07
Weight (kg)	79.9	8.3
Body Mass Index (kg·m^-2^)	24.1	1.4
Body Fat (%)	18.1	1.7

### Ad libitum feedings

Voluntary food intake was studied continuously for 16 weeks, whereby subjects consumed only foods provided by the Human Studies Facility (HSF) at the Beltsville Human Nutrition Research Center (BHNRC). Subjects choose foods *ad libitum *from the menus, and could consume any part or all of a food item, then return the remaining portion to be weighed. BHNRC staff that came into contact with the subjects provided no guidance as to the quantities and/or types of food items chosen. During weekdays, subjects reported to the BHNRC in the morning to eat breakfast, pack selected food items for lunch, then return again in the evening for dinner. Any food taken from the HSF that was subsequently not eaten (all or partial quantities), was returned the next day, and weighed and recorded. On Friday evenings, subjects were provided with coolers packed with a large amount of food for weekend meals. The weekend coolers provided a wide variety of foods in excess quantities, and subjects were allowed to request additional food items be included. Weekend food could be consumed on either day as long as the subjects logged which day each food item was eaten. All uneaten weekend food was returned on Monday, and weighed and recorded. Although subjects were instructed to consume only food items provided by HSF, they were allowed free access to beverages including caloric, noncaloric and alcoholic beverages. Detailed records of the amount, composition and name brand of beverages was submitted daily. In addition to beverages provided on the menu (milk and juice), both regular and decaffeinated coffee and tea were available at meals.

### Menus

Food items offered in the morning (breakfast and lunch) were presented in a cafeteria-style setting as three different rotating menus, each lasting seven days (Table 5). Some food items remained on all three menus (e.g. milk and orange juice). In the evening, breakfast and lunch items were also available. A typical dinner was presented cafeteria-style as one or two entrée selections with optional gravies or sauces, and a minimum of three vegetables and side dishes. A garden salad with a variety of additional toppings and dressings was also available. Fifteen different dinner menus were rotated daily (Table 5).

**Table 5:** Representative food offerings during breakfast and lunch (one of three weekly rotations), and one dinner (1 of 15 daily rotations).

**BREAKFAST AND LUNCH**							**DINNER**
Beverages	Cereals	Bread	Meat, Dairy, Eggs	Snack	Packaged Foods	Produce	#15
2 % milk	Hot (6)	English muffin	Ham	Fig bars	Vegetable soup	Apple	Turkey
Skim milk	Cold (10)	Waffle	Chicken salad	Granola bar (LF)	Beef w/veg soup	Orange	Chicken gravy
Orange juice		Honey bun	Salami	Popcorn	Clam chowder	Banana	Mashed potatoes
Apple juice		Bread (4)	Provolone cheese	Short bread cookies	Noodle soup	Grapes	Mixed
Vegetable juice		Pita bread	American cheese	Brownie	Pizza	Peaches	Citrus salad
		Buttery cracker	Scrambled egg	Strawberry twist	Pocket sandwhich	Dates	Cranberry sauce
		Saltine cracker	Bacon	Chocolate bar (2)	Sausage biscuit	Garden salad	Sourdough bread
			Yogurt (FF)	Peanuts		Lettuce	Macaroni & cheese
			Cottage cheese	Peanut butter		Tomato	
			Parmesan chesse			Carrots	
						Cucumber	
						Celery	

(#) = number of items available in a category

LF = low fat

FF = fat free

The goals of the menu design were to allow detection of macronutrient selection by offering a wide range of carbohydrate, fat- and/or protein-rich foods, and to provide a variety of commonly available foods typical of what many Americans eat. In a research setting it is impossible to duplicate the degree of food choice available in real life. However, more than 300 food items were used to develop menus for this study, and specific requests for food items were incorporated into the menus whenever possible.

### Recording and tracking of food intake

After each subject selected his desired foods, he presented them to a staff member that recorded the identity and weight of each food item by hand and on a computer (combination of bar code recognition of the food item and hand-entering of the weight). Upon termination of feeding, each subject presented his tray to a staff member that weighed any uneaten food. The accuracy of the food item recording process was verified by comparing the information on the computer with the hand-entered logs. This verification procedure was followed daily, and repeated at the end of the study with all food records. Energy and macronutrient composition were determined by consultation with the USDA Nutrient Database for Standard Reference [29].

### Covert manipulation of macronutrient composition

During the 16 weeks of *ad libitum *intake, subjects were randomly assigned to two of three treatments. Each treatment lasted 8 weeks with no break between the periods. The treatments consisted of a daily beverage that contained ≈ 2 MJ/day of predominantly carbohydrate (Hi-CHO), fat (Hi-FAT), or a combination of protein and carbohydrate (Hi-PRO) (Table 1). The daily beverage was divided into three equal portions, and subjects consumed them with each of the three primary meals. The protein drink was designed to provide half the daily Recommended Daily Allowance (RDA) [30] of protein, with the balance carbohydrate. The drinks were formulated using sucrose, heavy whipping cream, and egg white as the principle source of carbohydrate, fat, and protein, respectively. Water, fat free non-dairy creamer, and aspartame were used to provide volume, adjust texture and add sweetness. Cocoa was added to all drinks to provide a uniform taste and appearance. Subjects were blinded to the treatments and the three drinks were judged to be indistinguishable by a taste panel conducted in our laboratory.

### Ghrelin analysis

Each morning for the last seven days of each treatment period, subjects reported to the laboratory after a 10–12 hr fast, provided a blood sample, then reported to the HSF to eat breakfast. Blood was collected in tubes containing EDTA, centrifuged, and stored at -80°C until analysis. Plasma ghrelin was analyzed using a commercially available radioimmunoassay kit (Total Ghrelin, Linco Research, Inc.). The intra- and interassay coefficients of variation (CV) were 5.6% and 7.3%, respectively.

### Body weight and composition

Before breakfast and after voiding, body weight was determined weekly on an electronic balance to the nearest 0.01 kg. Body composition was measured by Dual-energy X-ray Absorptiometry (DEXA; QDR 4500, Hologic, Inc, Waltham, MA).

### Total and 24 hr energy expenditure (24EE)

To "capture" daily variations in energy expenditure, we combined a self-reported activity log [31] and doubly labeled water measurements. Although doubly labeled water is the "gold standard" measure of free-living energy expenditure, its use is limited by the production of a single value that is assumed to represent average energy expenditure over the course of the dosing period (seven days in this study). This seven day value for energy expenditure is not useful to compare with daily variation in ghrelin and food intake (macronutrient composition and energy intake). Since self-reported measures of energy expenditure (that can provide a daily energy expenditure value) may be misreported by subjects [32,33], we adjusted the daily numbers using doubly labeled water measurements (see below).

Twenty-four hour energy expenditure (24EE) was estimated using a daily recording log method, modified from Bouchard et al. [31]. Briefly, subjects recorded their daily activities in a log every 15 min over the course of the last seven days of each treatment period. Activities were entered in as a number (1–9), corresponding to example activities listed in the log. Each activity assumed a pre-determined energy expenditure score, thus energy expenditure was calculated as time spent in that activity times the energy expenditure rate.

Total energy expenditure (TEE) was concurrently measured by the doubly labeled water method as described by Speakman [34], which provided an estimate of energy expenditure during the last seven days of each treatment period. Subjects reported to the BHNRC between 6:30 and 9:00 a.m., at which time they received an oral dose of H_2 _^18^O (0.16 g/kg body weight) and ^2^H_2_O (0.30 g/kg body weight). Urine samples were collected immediately before the dose and on every morning (second void) for the last seven days of the treatment period. The first sample was collected approximately 24 hr after the dose. Enrichments of ^2^H and ^18^O in urine samples were measured by infrared spectroscopy and isotope ratio mass spectrometry, respectively. TEE was calculated using the equations of Weir [35].

Individual daily 24EE values were corrected using the ratio adjustment (notation denoting subjects is suppressed),

24EE_dayx, corrected _= 24EE_dayx _× (TEE/24EE_day 1–7_), where

24EE_dayx _is the uncorrected daily energy expenditure value from the activity log for one of the seven days (day X),

TEE is the daily mean energy expenditure estimate using doubly labeled water. Represents a single value during the seven days of measurement (of which 24EE_dayx _is one), and

24EE_day 1–7 _is the mean of the seven days of uncorrected 24EE values corresponding to TEE, of which 24EE_dayx _is one.

To simplify the notation, the 24EE_dayx, corrected _value for day X will subsequently be referred to as 24EE.

### Data transformation

To check the assumption of homogeneous variances necessary for valid F-tests and correct P-values, we used the standard technique of plotting the standard deviations (SD's) against the means for selected energy intake, grouping observations by subject and treatment period. The results of this scatter plot revealed a strong positive linear relationship (r = 0.67, P < 0.001). The relationships between the SD and mean for macronutrient and energy intake (total and selected), and 24EE were also positive and significant. This indicated that the SD's (variances) were a function of the mean and that the data needed to be transformed. We followed methods described by Draper and Smith [36], and used a family of transformations based on logarithms. For selected energy intake, this transformation was log (b_0 _+ b_1_y_i_), where b_0 _and b_1 _are the estimated coefficients of the line fit by regressing the SDs on the means, and y_i _represents the energy intake data. The other variables were transformed using this same family of transformations. This procedure resulted in homogeneous variances for all variables once transformed, satisfying ANOVA assumptions. We present the data on the original scale in tables and figures for ease of interpretation (unless indicated otherwise).

Due to the free-living nature of the subjects, there were three observations (of 168) where (for unknown reasons) a subject's food intake differed greatly from habitual intake due to a skipped meal or meals with low energy intake. For this reason, these observations were not used in the analyses. Additionally, a preliminary sensitivity analysis and residual diagnostics (e.g., restricted likelihood distance, Cook's D; optional output of Proc Mixed, new in version 9.1, in [37]) suggested they were outliers.

### Statistical analysis

The experimental design was an incomplete block crossover design, with two of the three drink treatments given sequentially to each subject. Data were analyzed in the mixed linear models framework, using the Proc Mixed procedure in SAS (version 9.1)[37]. Subject-to-subject variation was modelled as a random effect. Repeatedly measuring each subject over the seven days induced an autoregressive covariance structure we modelled as AR(1). Other design effects we retained in our modelling were a two level period effect ((first 8 week treatment period (1) vs. the second 8 week period (2)), and two day-of-the-week variables, found in a preliminary analysis to account for day-of-the-week effects. Each of these day-of-the-week variables classify days into two groups: (1) Sunday (0 vs. 1 for other days of the week) and (2) Monday/Friday (0 vs. 1 for other days of the week). They allow for the major differences in food intake and energy expenditure due to day-of-the-week effects. Some subject-specific variables, such as body weight, were included as covariates as appropriate. The treatment effects (Hi-CHO, Hi-PRO, and Hi-FAT) were included in all models.

For models predicting ghrelin concentration, we included 24EE, energy intake, and the interaction between 24EE and energy intake. We also considered prior day (up to 7 days) and subsequent day (up to 2 days) values for energy intake and ghrelin, and their interactions as candidate covariates. Values for up to 4 prior days for 24EE were used to predict ghrelin. For models predicting daily energy intake, we included preprandial ghrelin concentrations, 24EE, the interaction between ghrelin and 24EE, and additionally considered as candidate covariates the prior (seven days) and subsequent (two days) days for these two variables and their interactions. We explored models that included other variables and interactions, but none of those variables appeared useful. Data are presented as total intake (intake including treatment drinks) and/or selected intake (intake without treatment drinks). Values are presented as means ± SD unless indicated otherwise.

Since a preliminary analysis suggested that the effect of ghrelin on energy intake was small or negligible, we conducted a power analysis to determine our ability to detect an effect of ghrelin if the effect was small. This was accomplished by Monte-Carlo simulation (creating simulated data sets based on the data we collected) and, starting with no effect of ghrelin (a true coefficient of zero for ghrelin in a regression context), determining how large the true coefficient needed to be to obtain significance for most of the simulations, at powers of 80%, 90%, and 95%, with 1000 simulations for each coefficient value. These results are most easily interpreted as how large a partial correlation between ghrelin and energy intake (adjusting for all other fixed and random effects, other than ghrelin) would be necessary for us to detect it. We conducted this analysis for both total energy intake and breakfast energy intake (the latter was the meal most likely to be influenced by preprandial ghrelin because of the timing of the blood draw).

## Authors' contributions

MK was responsible for statistical analysis and interpretation. DR was responsible for supervising the food intake portion of the study. WR conceived the study, and supervised the data collection and analysis. DP was responsible for ghrelin analysis, data collection, statistical analysis and manuscript preparation. All authors read and approved the final manuscript.
